# Public engagement, evidence review and survey to adapt a whole-school intervention to prevent bullying in English primary schools

**DOI:** 10.3310/nihropenres.13975.2

**Published:** 2026-05-05

**Authors:** Rose Eagle-Hull, Neisha Sundaram, Miranda Perry, G.J. Melendez-Torres, Chris Bonell

**Affiliations:** 1 London School of Hygiene and Tropical Medicine Department of Public Health Environments and Society, London, England, UK; 2 Independent Consultant, London, UK; 3 Department of Public Health and Sports Sciences, University of Exeter, Exeter, England, UK

**Keywords:** school; bullying; children; patient and public involvement and engagement; intervention adaptation

## Abstract

**Background:**

Bullying increases during primary school and causes multiple mental/physical health harms. Whole-school interventions offer a feasible means of reducing bullying but few have been evaluated in primary schools. We previously trialled the Learning Together intervention in secondary schools comprising local needs assessment, student and staff participation in decision-making through ‘action groups’, restorative practice, and a social and emotional skills curriculum. This intervention was effective in preventing bullying and improving mental wellbeing. We aimed to adapt Learning Together for primary schools (Learning Together Primary Schools (LTPS)). This paper reports on how we adapted intervention materials to produce the LTPS intervention through a review of research evidence, online survey, and patient and public involvement and engagement (PPIE).

**Methods:**

We conducted a rapid review of existing systematic reviews, online survey of primary schools in south-east England, and multiple PPIE workshops. PPIE was conducted with two primary schools (10 staff members and 20 pupils), with a group of 10 pupils from five primary schools, and with a group of six parents with primary-school-aged children.

**Conclusions:**

We refined our initial plans for LTPS, developing an intervention appropriate for primary schools and supported by full materials, training and external facilitation. We retained key components including restorative practice and action groups and made several refinements, including guidance for action group implementation to accommodate for primary schools’ smaller capacities. No refinements were made to the intervention theory of change. We found that it is possible to refine and elaborate interventions to provide full materials and support via processes drawing on evidence review, a survey and PPIE. Although not all PPIE suggestions could be acted upon, PPIE proved valuable in ensuring the feasibility and acceptability of the intervention in primary schools. Future work will include a pilot trial to assess whether progression to a full trial is justified. Study registration ISRCTN10215449 https://doi.org/10.1186/ISRCTN10215449

## Plain Language Summary

Bullying is experienced by many children in England and can cause mental and physical health problems. Schools can help reduce bullying through activities that aim to change how the school is run, and by improving relationships in the school. We previously evaluated an intervention called ‘Learning Together,’ which used this approach to prevent bullying in English secondary schools. It asked students about their needs and involved students and staff in decision-making. It also trained teachers so they could address bullying and conflict by focusing on repairing relationships rather than simply punishing perpetrators. It was effective in preventing bullying and also improved students’ wellbeing. In the present study, we adapted Learning Together to be used in primary schools, calling the new intervention ‘Learning Together Primary Schools’. To refine each part of the Learning Together intervention for Learning Together Primary Schools, we did several things. We reviewed existing research to find out the best ways to address bullying in primary schools. We sent an online survey to primary schools in south-east England to check whether schools currently address bullying using similar approaches. We also worked with staff and pupils from primary schools, and with parents with primary-school-aged children to ask their views on specific parts of the intervention. We did this to make sure it would be possible to introduce the adapted intervention in primary schools, and to make sure that all components were acceptable for both staff and pupils. Advice from school staff, pupils and parents helped make the intervention more practical, acceptable and inclusive for primary schools. We look forward next to trying out the adapted intervention in schools to check that it is possible for schools to deliver.

## Introduction

This paper reports on the adaptation for English primary schools of ‘Learning Together’, a ‘whole-school’ bullying-prevention intervention found to be effective in a randomised controlled trial (RCT) in English secondary schools. ^1^ ‘Whole-school’ refers to going beyond just delivering classroom education to promote health by modifying the school environment, including how staff and pupils interact with each other what shared beliefs are held. ^2^

Bullying is repeated behaviour by an individual or group that intentionally hurts another physically or emotionally. ^3^ In England, among 11 year-olds, a third report victimisation in the previous two months with a sixth reporting cyberbullying victimisation. Bullying increases with age during primary school and is more common among disadvantaged students. ^4, 5^ Bullying causes multiple physical/mental health harms in childhood and adulthood, ^6– 11^ and lower educational attainment. ^12^ Schools often use punitive approaches ^13^ despite evidence these are ineffective. ^14^ An alternative approach, restorative practice, aims to prevent or resolve conflicts between/among students or staff. ^15^ Victims communicate the impact of harm. Perpetrators acknowledge and amend their behaviour. Restorative practice involves primary prevention via use of restorative language, ‘circle-time’ (gatherings to discuss feelings and relationships to prevent conflict before it arises) and/or secondary prevention via conferencing (parties in conflict develop strategies to avoid future harms). There have been repeated calls that bullying-prevention is a priority for which evidence is required, including on the effectiveness of restorative practice in primary schools. ^3, 16, 17^ It has been suggested that restorative practice is most effective when done on a whole-school basis, for example coordinated by an action group comprising staff and students. ^18^

A recent Campbell Collaboration systematic review of school-based bullying prevention reported effectiveness in reducing perpetration (OR = 1.31: 95% confidence interval (CI) 1.24, 1.38) and victimization (OR = 1.24: 95% CI 1.19, 1.31). ^19^ The review included no RCTs of restorative practice in primary/elementary schools but three quasi-experimental studies of restorative practice in these settings, reporting mixed findings. Recent systematic reviews focused on school restorative practice were poorly conducted and lacked meta-analyses, but included two US RCTs of restorative practice among primary-school-aged children. ^20– 22^ An RCT of ‘Safer Saner Schools’ (whole-school restorative practice) reported reductions in school suspensions. ^23^ An RCT of another restorative practice intervention reported reduced bullying victimisation among those reporting exposure to the intervention but no overall intention-to-treat effect. ^24^ These systematic reviews of restorative practice conclude, largely on the basis of quasi-experimental studies, that it is effective in reducing bullying among primary school aged children. ^20– 22^ Restorative practice is likely to be more feasible to implement in primary schools than are curriculum-based interventions because restorative practice does not require classroom learning time for delivery. Curriculum-based bullying prevention has proven challenging to deliver with fidelity in the UK and elsewhere because of lack of space in school timetables. ^25, 26^

A previous RCT examined the Learning Together intervention in English secondary schools. This was a multi-component, whole-school intervention aiming to modify the school environment to reduce bullying. The key elements were: survey of students to identify needs; action groups comprising staff and students (supported by an external facilitator) to review needs data and use this to plan and coordinate local delivery, and revise school policies and rules; training for school staff in restorative practices; and a social and emotional learning (SEL) classroom curriculum. The trial found that the intervention reduced bullying victimisation (primary outcome) as well as social and emotional problems, improved mental wellbeing, improved health-related quality of life, and reduced substance use. ^1^ The intervention was highly cost-effective. ^27^ The SEL curriculum was poorly implemented and so could not account for these impacts.

We aimed to adapt ‘Learning Together’ for primary schools (termed ‘Learning Together Primary School’ (LTPS)) prior to a pilot RCT which would assess feasibility and acceptability of the intervention and planned intervention design. Early intervention in primary schools is likely to have greater impacts than when delivered in secondary schools given recent evidence that bullying prevention is more effective among younger students. ^28^

Careful and systematic adaptation of existing effective interventions is generally more efficient than developing new interventions. ^29, 30^ However, because complex interventions interact with contextual factors in various ways, they may have limited effectiveness or even cause harm when poorly adapted to new contexts. ^30– 32^ Recent adaptation frameworks emphasise: defining the problem to be addressed; identifying and building on an evidence base for the intervention being adapted; exploring differences between the original and new context, and how these might affect implementation and intervention mechanisms; assessing potential unintended consequences of adaptation; and iterative involvement of stakeholders. ^29, 31, 33^ The ADAPT guidance offers a reporting checklist for adaptation consisting of describing the: problem being addressed; original intervention, its context and evidence base; new context including similarities and differences to the original context; rationale, type, and processes undertaken to adapt the intervention (including which stakeholders were involved); adapted intervention in detail to enable replication; and role of original intervention developers in adaptation. ^29^

A central element of intervention adaptation is patient and public involvement and engagement (PPIE), which aims to consider the views of service users, citizens and practitioners with lived experiences. ^34^ These stakeholders have in-depth understandings of local context that can illuminate whether interventions are likely to be feasible and acceptable. However, effective PPIE is complex and challenging, with a risk that processes are tokenistic and participant views are marginalised. ^35^

Our adaptation to produce LTPS was informed by the above frameworks and involved PPIE with primary schools plus rapid review of existing systematic reviews and a survey of primary schools. It aimed to examine the following questions:

1) Do systematic reviews suggest there are intervention components that are elements of effective primary school bullying-prevention interventions, which are consistent with the theory of change for Learning Together, and which could be feasibly incorporated into LTPS?
2) Do systematic reviews suggest there are factors influencing the feasibility, acceptability, reach or effectiveness of primary school bullying-prevention interventions that should be considered in our adaptation?
3) What does in-depth PPIE suggest about how primary schools differ from secondary schools and so how Learning Together should be adapted?
4) What does a wider survey of primary schools suggest about existing bullying-prevention and related provision, and the feasibility, acceptability and the acceptable pricing of the intervention activities under consideration for inclusion in LTPS?

## Methods

### Patient and Public Involvement

As per NIHR open research journal guidelines, patient and public involvement is briefly summarised here. Previous patient and public involvement and evaluation (PPIE) conducted with senior leaders and teachers from primary schools in South-East England suggested that Engllish primary schools lack effective bullying prevention interventions and are receptive to an approach involving action groups and restorative practice. Their expertise and priorities underly our aim to adapt an effective bullying prevention intervention. This paper reports in detail on how PPIE informed the adaptation of the Learning Together Intervention for primary schools in the methods section below, “ *Patient and Public Involvement and Engagement (PPIE) with primary schools and parents*”. The choices made following public involvement in the intervention design and conduct are reported in the results section below, “ *PPIE with primary schools and parents*”. Through these workshops, the public was involved in shaping and refining the Learning Together Primary Schools intervention, prior to a pilot RCT that will aim to assess the feasibility and acceptability of the intervention.

### Overall design

The adaptation was conducted from December 2023 to September 2024 and comprised: rapid review of systematic reviews of whole-school and restorative practice interventions in primary schools; PPIE with primary school staff, pupils and parents; and an online survey of primary schools in south-east England.

### Initial plans for intervention

Adaptation of Learning Together for primary schools drew on the original Learning Together intervention and its materials. Our initial plans for intervention were described in our funding application for the adaptation and pilot study. At the proposal stage, informed by initial consultation, it was decided that LTPS should be informed by a similar theory of change to Learning Together and include most of the same components: a survey of pupils to identify needs; action groups comprising staff and pupils (supported by an external facilitator) to review needs data and use this to plan and coordinate local delivery, and revise school policies and rules; and training for school staff in restorative practices.

Consultation with senior leaders and teachers from five primary schools in south-east England to inform the funding proposal indicated that schools were enthusiastic about restorative practice. While some were already using this, staff reported lacking training and support materials. School leaders suggested that most schools already delivered SEL and would struggle to implement additional contents due to timetabling space. We were therefore unsure whether to include an SEL curriculum and so this was a question to explore in the adaptation phase. Other components were retained with a view to adapt as necessary. For example, given the age of primary school children, we intended to explore whether they would engage in action group discussions and find restorative practice acceptable.

**Figure 1.**
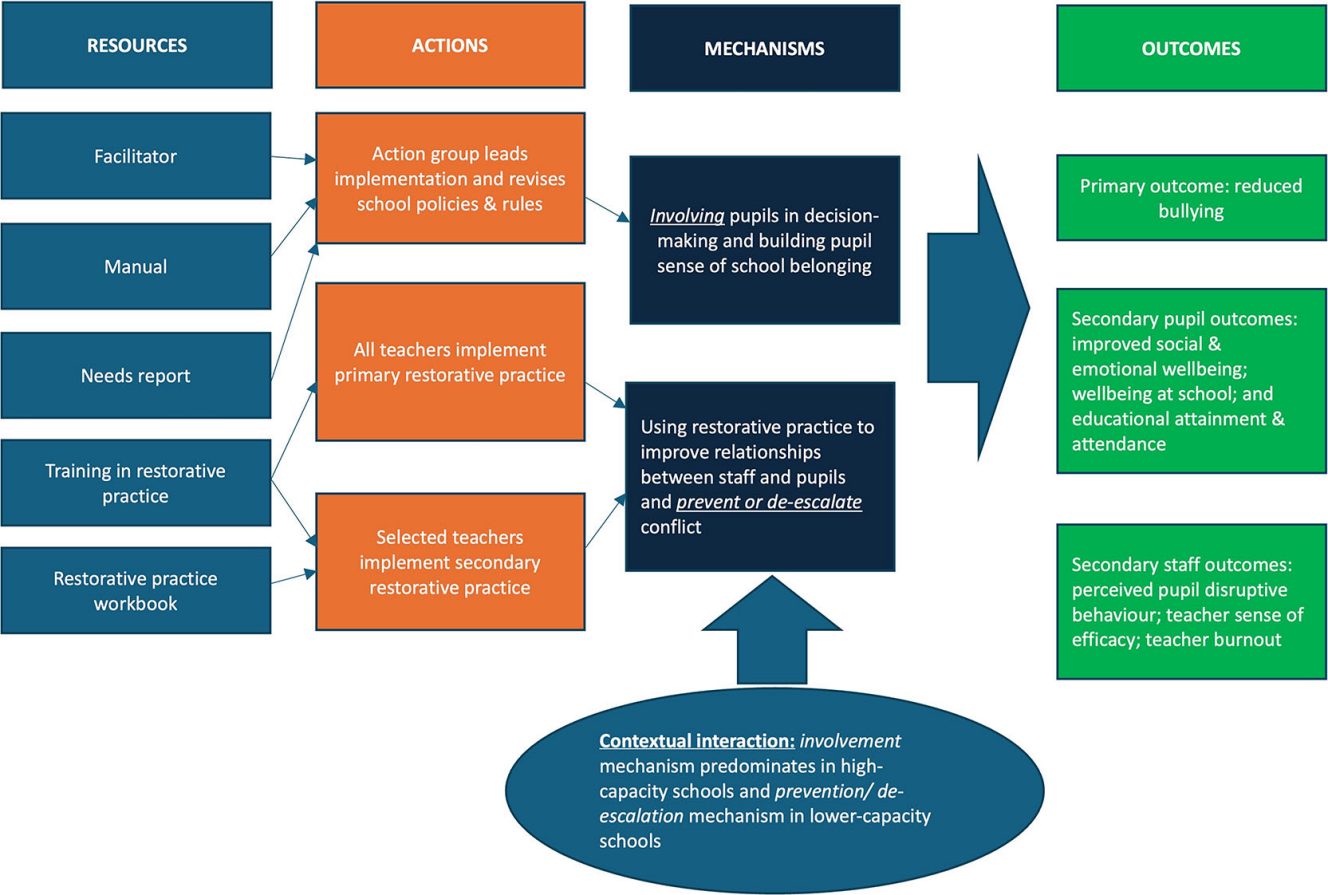
Logic model of LTPS theory of change.

***Theory of change.*** LTPS’ theory of change, like that of the original Learning Together intervention, was informed by the theory of human functioning and school organisation. ^36^ Learning Together aimed to reduce bullying, and social and emotional problems via two mechanisms: using an action group to involve students in school decisions and build school belonging; and using restorative practice to improve relationships and prevent/de-escalate conflict ( Figure 1). These are theorised to together bring about reductions in bullying as well as improve social and emotional wellbeing, wellbeing at school, and educational attainment and attendance. They are also theorised to bring about staff benefits in terms of reduced perceived student disruptive behaviour, increased teacher sense of efficacy and reduced teacher burnout.

***Materials.*** Schools were to receive an anonymised report on overall pupil needs (drawing on baseline survey of pupils in years 2–6 aged 6–11 years), an intervention manual and a workbook to help teachers run restorative practice sessions. As reported above, initial PPIE to inform the funding proposal suggested primary schools already delivered SEL and would struggle to deliver additional contents. Therefore, further PPIE in the adaptation phase explored whether to include a curriculum component.

***Training/facilitation.*** All school staff were to receive a two-hour introductory training in restorative practice. Around four to five selected staff per school would receive a three-day in-depth training provided by L30 Relational Systems. The Place2Be mental health charity would facilitate action groups.

***Procedures, delivery & dose.*** The intervention was to be led by a school lead in each school and a more senior staff-member who would chair the action group. All staff were to undertake restorative practice in their classrooms to prevent conflict and respond to minor incidents coming to their attention. Selected staff would facilitate restorative practice conferences in response to more serious incidents. The action group would meet termly and: review needs data to decide local priorities; revise rules/policies to support restorative practice; and plan communications to pupils/parents. The action group would include approximately six staff (diverse by seniority/role) and six pupils from years 2–6 aged 6–11, also diverse by gender, ethnicity, school engagement. If it was determined in the adaptation phase that the intervention should involve a curriculum, schools would deliver this in lesson time.

***Planned adaptations.*** Guided by the manual and needs data, schools would identify local priorities, for example in terms of the forms of bullying to be addressed and the pupil groups most affected, to inform their provision.

### Rapid review of systematic reviews

This was informed by existing guidance for systematic reviews of reviews but did not aim to be a comprehensive systematic review. ^37^ Rather, it aimed to rapidly review systematic review evidence to inform decisions about whether other intervention components should be added (Q1) and what adaptations might be appropriate to ensure the intervention was feasible and acceptable in primary schools (Q2). The review had the following inclusion criteria: systematic reviews of bullying-prevention interventions; focusing on interventions targeting pupils of primary-school age; including a focus on whole-school interventions or restorative practice to prevent bullying; including RCT or quasi-experimental studies with no treatment, usual treatment or other treatment control groups; focusing on bullying outcomes; and reported in English.

We conducted searches of the Education Resource Information Centre, PsycINFO and Pubmed bibliographic databases. We took the following concepts from our inclusion criteria to develop a search string: school [MeSH] AND bullying [MeSH] or cyberbullying [MeSH] AND systematic review [pt] or evidence synthesis. Because this was a review of reviews, we could include a term for bullying without this potentially introducing publication bias. Additionally, we included some reviews of which we were already aware and asked several experts for additional suggestions (Appendix 1).

We extracted data on reviewer description of: components of interventions identified as significantly effective among children aged 5–11 according to RCT or quasi-experimental studies; what factors influence the feasibility, acceptability, reach or effectiveness of primary-school bullying-prevention interventions (Appendix 2). We did not assess the quality of the systematic reviews. In synthesising findings, we assessed whether the components of effective interventions reported in reviews involved a theory of change that involved, or was compatible with, the de-escalation of conflict or building of pupil sense of belonging, and, if so, whether these could plausibly be incorporated into the LTPS intervention. We also assessed whether review findings about the factors that might influence the feasibility, acceptability, reach or effectiveness of interventions should inform the design of LTPS intervention.

### Patient and Public Involvement and Engagement (PPIE) with primary schools and parents

We adapted LTPS in collaboration with staff and pupils from two primary schools, plus a group of children recruited from five other primary schools, and a group of parents. PPIE explored how primary schools differ from secondary schools and therefore how Learning Together should be adapted (Q3). As this was PPIE and not research, formal consent was not required and sessions were not audio-recorded. Staff and pupils were given verbal information about the workshops and freely agreed to participate. We took written notes during the workshops. In each workshop, PPIE members were guided by slides to review existing Learning Together materials and successive drafts of adapted materials.

We held three in-person, 90-minute workshops at each of two primary schools in south-east England between January and April 2024 (with around 45 minutes involving staff and 45 minutes involving pupils). We aimed to involve schools with free-school-meal entitlement above the 2023 national average of 27.1% (indicating high pupil need) and a national-school-inspectorate rating of good or outstanding (indicating school capacity to collaborate). These were recruited by emailing existing contacts, including schools that had previously indicated interest in participating in research. These schools received £400 compensation. The two schools were asked to select 10 pupils from years 5–6 (diverse by gender, ethnicity and school engagement) and five staff-members (diverse by role/seniority). Pupils received a certificate to thank them for their involvement.

During the first workshops, pupils were presented with the overall components of LTPS and asked to discuss action groups and the needs survey. In the second meeting, they were asked about their views on restorative practice, including whether they thought it was interesting and useful, what they thought of it in comparison to punitive approaches and whether their school already implemented restorative approaches. In the third workshop, pupils were asked their views on the proposed manual, needs report and communication materials about LTPS for pupils.

In the first staff workshop, staff were given an overview of the intervention and asked whether they already implemented restorative approaches. They also discussed the SEL curriculum, action groups, pupil-led revision of school rules and the needs survey. In the second meeting, staff were asked their views on the proposed training for restorative practice, and on communicating about LTPS to the rest of the school and parents. In the final workshop, staff were asked their views on the role of the day-to-day lead, on action group scheduling and communication, the needs report and the manual.

We also held two 60-minute online meetings with a group of children recruited in pairs from five other primary schools in south-east England in July 2024. These schools were recruited by emailing existing contacts and were compensated £100 per meeting. Pupils received a certificate to thank them for their involvement. Schools were asked to recruit relatively disengaged pupils. The pupils were accompanied by a teacher from their school. They were asked their views on restorative practice and action groups (including interest in participating in an action group, communication materials and activities, and decision-making with teachers and pupils from other year-groups). They were also asked about the needs report.

Finally, we held a 60-minute, in-person meeting with a group of parents of primary-school-aged children in July 2024, via existing contacts with primary schools in south-east England. Parents were compensated with a £10 voucher. Parents were asked about their views on emotional literacy, restorative practice, action groups and bullying.

After the workshops, PPIE members’ views and suggestions were discussed within the research team. Suggestions were taken on board as far as possible, provided they were feasible and did not contradict existing evidence.

### Online school survey

We also conducted a short survey of schools in south-east England (Berkshire, Buckinghamshire, Essex, Hertfordshire, Kent, London, Surrey) using onlinesurveys.com (Appendix 3) to explore existing bullying-prevention and related provision, and the feasibility, acceptability and the acceptable pricing of the intervention activities under consideration for inclusion in LTPS (Q4). Information and a link to the survey were sent to administrative email addresses of 3687 schools with the request that this be forwarded to a staff-member responsible for behaviour, pastoral care or bullying prevention, with a reminder one week later. Unlike the other components reported here, the survey was treated as research and approved by LSHTM ethics committee (reference 29958). The email sent to schools presented information about the study and the survey, and potential participants were asked to read this and indicate their consent before being taken to the survey questions. Descriptive analysis using Stata (StataCorp LLC, Version 15) was used to calculate frequencies.

## Results

### Rapid review of systematic reviews

Appendix 4 reports the references identified by our searches. We included 21 studies. ^19– 21, 38– 54^ Appendix 5 provides detailed information about what information we extracted from each review. The review of systematic reviews provided some useful results in terms of informing how we might adapt and optimise Learning Together for primary schools. This was despite gaps in what systematic reviews reported. One of the included reviews did not report on intervention effectiveness at all. ^21^ Six reviews did not report any effective interventions delivered in primary or elementary schools. ^20, 38– 41, 50^ Another review reported no effective interventions for primary schools where the theory of change centred on prevention of bullying via de-escalation of conflict or increasing pupil belonging (as is the case with Learning Together). ^42^ Most reported limited details of effective interventions. Few reported on implementation or the factors affecting this. As a result, these reviews offer limited insights into how to adapt Learning Together for primary schools.

In terms of potentially effective intervention components, evidence from the reviews suggested the usefulness of multi-component interventions. ^38^ Four reviews identified the effectiveness of using restorative practice or other ways of working with pupils involved in bullying to prevent this behaviour. ^19, 43– 45^ Some reviews also suggested the potential value of other components including: peer mediation ^46^; developing or refining school policies on bullying or discipline ^19, 44, 45, 47^; parent meetings or other communications ^19, 44, 48^; increasing staff presence and visibility in hot-spots for bullying ^44, 45^; rewarding pupils for good behaviour ^44^; and enhancing pupil breaktime activities. ^44, 45, 47^ Four ^19, 43– 45^ of the interventions reported to be effective included classroom SEL lessons. There was no evidence that SEL lessons were an essential element of effective interventions.

In terms of the factors likely to promote implementation, some reviews noted the importance for implementation of building support from school leaders ^44, 45, 48^ including by ensuring interventions aligned with national policy requirements. ^48^ Reviews also noted the value of supporting intervention with staff training, ^19, 43, 45, 46, 48^ and providing a clear and accessible manual for schools. ^44^ Several reviews reported that multi-component interventions are more feasible and acceptable when it is clear how the components are synergistic. ^44, 48, 49^ Several reviews highlighted the value of using staff/pupil action groups as a means to tailor and coordinate implementation to local contexts ^44^ and of providing external expert facilitation for these meetings. ^45, 48^ Some reviews also noted the value of using data on pupil needs to inform tailoring of provision. ^44, 48^ There was evidence that pupil involvement on action groups was likely to be particularly successful when pupils were given clear roles with the opportunity to express themselves. ^44^ In terms of restorative practice or conflict resolution, there was evidence that restorative practice was more feasible and acceptable when tailored to school context via prior assessment of the characteristics of the school/community. ^21, 44^ There was also evidence that involving the classroom teachers of pupils involved in bullying or other conflict could be part of an effective approach to teachers working together to de-escalate this. ^43^

### PPIE with primary schools and parents

***In-person workshops with primary schools.*** Two mixed-sex state primary schools were selected, both matching our criteria of having an above-average free-school meal-entitlement rate and good inspection rating. School A was an academy-sponsor-led school with over 170 pupils and school B, a community school with over 300 pupils. Both had free-school-meal eligibility of nearly 40%.

Table 1 reports pupil demographics and Table 2 staff roles.

**Table 1.** Pupil demographics.

School	Year-group				Gender	
	*3*	*4*	*5*	*6*	*Girls*	*Boys*
A *	0	0	3	7	4	6
B	3	2	3	2	7	3

^*^ *In the second workshop, one year-five pupil was absent and in the third workshop, five year-six pupils and one year-five pupil were absent.*

**Table 2.** Staff demographics.

School	Role					
	*Teacher*	*Teaching assistant or learning support assistant*	*Head-teacher *	*Assistant head*	*Administrative*	* Emotional literacy support assistant*
A	1	1	1	0	1	1
B	1	3	0	1	0	0

***School A***

We first met with pupils from school A on 23 ^rd^ January 2024. Pupils were enthusiastic about the overall programme. They found the needs survey easy to understand. However, several pupils stated that year-two pupils should not be asked questions about bullying. They were concerned that the bullying definition was too long and contained complex vocabulary, e.g., ‘exclude’ and ‘rumours’, which they thought younger pupils might not understand. Pupils were in favour of action groups. However, they suggested meetings more than once per term would be preferrable. Pupils supported the idea of the needs data being presented to action groups in anonymised form. All pupils welcomed the idea of pupils being involved in revision of school rules and contributing to decisions.

In the second workshop, on 18 ^th^ March 2024, most pupils were unsure whether their school already employed restorative practice, but were positive about this approach when it was explained. Several year-five pupils thought it might make children kinder and reduce feelings of anger or sadness that might arise when they were reprimanded. Pupils thought that speaking directly and calmly with pupils could improve relationships among classmates and teachers. However, two year-six pupils were concerned that some children might not take restorative practice seriously or might not understand how another child feels.

In the third workshop, held on 28 ^th^ March 2024, when shown an action group poster that schools could use to recruit pupils, pupils thought that younger pupils would struggle to understand terms such as ‘diverse’. However, once this was explained, pupils supported action groups being diverse and thought that those struggling academically could benefit from joining. Pupils thought that action group members could communicate its purpose in simple language to their peers. They wondered whether shy pupils might not want to speak up, but thought encouraging pupils to work and discuss in pairs before summarising their ideas in front of the group might help. Pupils liked the idea of the needs report informing actions. When shown a sample report, some pupils thought that year-three pupils would not understand percentages but could gain an understanding by looking at other numbers. Most agreed that older pupils would benefit from looking at percentages. Pupils suggested providing a summary of the information and a key to explain that ‘N’ referred to numbers, which they had not initially understood. Regarding the intervention manual, the group agreed that it should be circulated to all action group members, including pupils, providing it was written so that all year-groups could understand it. Pupils emphasised that it was important for younger pupils to feel included and that teachers should explain parts if necessary.

In the first staff workshop at school A, staff voiced support for a whole-school approach using restorative principles, and thought the overall intervention inputs sounded feasible and acceptable. They said they used restorative approaches but that this was piecemeal and not supported by any training. Staff wondered whether a one-year window would be sufficient for pupils on an action group to be involved in revision of school rules. They found the needs survey too long for year-two pupils’ concentration, and thought that some would answer randomly or not feel comfortable to answer at all. They thought the overall content was acceptable for year-three pupils and above. Staff confirmed that they already had an SEL curriculum, as would most other primary schools. They indicated that implementing an additional curriculum would be overwhelming for most schools.

At the second workshop, staff discussed the restorative-practice training, agreeing that an online format was acceptable. They confirmed that it would be feasible to allocate all staff to a two-hour introductory session. However, the headteacher suggested that because most primary-school staff have a full-time teaching commitment, schools, especially one-form-entry schools (which are relatively small with approximately 200 pupils), would struggle to release four or five staff for the in-depth training. Staff suggested that running the training on three non-consecutive days could ease this concern, adding the training would not have to fall on an in-service education and training day. They added that this would allow them to engage staff across each key-stage and enable useful staff dialogue, and thought that most other primary schools would likely manage this too. In terms of communicating about the intervention to parents, staff emphasised the importance of a letter for parents that demonstrated the potential benefit of the intervention to the school. They suggested that the letter should be clear that the needs survey is related to how pupils feel at school, rather than at home. They also suggested producing a video for parents.

At the final workshop, staff were sceptical about whether schools would have the capacity to provide one staff-member to chair the action group and one to be day-to-day lead, suggesting that one person could do both. Staff also emphasised the importance of maintaining good communication between the school and facilitator. The group agreed that pupils on the action group should be encouraged to lead assemblies for their peers. Staff thought pupils would manage with minutes-taking if supported by an adult, a useful skill for them to develop. They suggested displaying action group minutes alongside general information about the intervention on noticeboards. Staff did not think it appropriate to share the manual with pupils because this would be too long and detailed. Staff confirmed that holding termly meetings was feasible but proposed an additional interim meeting without the facilitator to help maintain momentum. Staff thought that pupils would understand the needs report but suggested including pie charts as visual representations. They said that some data were sensitive and queried whether pupils needed to see wellbeing data disaggregated by ethnicity or free-school-meals eligibility. Several staff suggested schools could decide which data they were happy to share with pupils. One participant was unsure whether pupils would understand or be comfortable with the term ‘ethnicity’. After discussion, the group agreed that, if presented carefully, this should not cause problems and might even enable thoughtful, compassionate discussion. Finally, staff indicated the importance of dedicating an entire meeting to reviewing the data.

***School B***

We first met with pupils from school B on 29 ^th^ January 2024. This school included some younger pupils in the group. In the first workshop, pupils voiced support for action groups. Younger pupils had mixed views about whether year-two pupils should be included. Regarding the needs survey, pupils thought most questions were easy to understand and answer for all year-groups. Year-three pupils agreed but thought some questions were too wordy. They suggested removing words such as ‘exclude’, ‘rumours’ and ‘ignore’.

The second workshop was held on 26 ^th^ February 2024. Some pupils agreed their school already used restorative practice and all thought it better than punitive approaches. They thought encouraging pupils to understand why conflict occurred would mean they were less likely to repeat such behaviour.

In the final workshop on 25 ^th^ March 2024, pupils agreed they would want to join an action group if their school implemented one. They thought it important to consider everyone’s views in order to contribute to school decisions and make a change. When shown the recruitment poster, pupils suggested making it more fun with shorter, positive sentences such as “you can change schools!”. Pupils thought they would be happy contacting their teacher to sign up, but that the poster should include practical information, such as meeting duration, time and location. Pupils agreed that group diversity was essential. A few were concerned that if too ‘grown-up,’ the groups might appear scary and prevent less-confident pupils from joining. Most supported the idea of group members telling other pupils about the work in assemblies. A few were concerned about feeling pressured to speak publicly and worried about making mistakes. The group agreed that it would be preferable for pupil and adult group-members to run assemblies together to reduce such pressure. When shown a sample needs report, pupils did not initially understand the data, which they thought were too complicated. However, after being talked through the tables, they agreed that an adult could explain them to pupils. They suggested including visual information, such as pie charts. Most thought that pupils on the action group should receive the manual if this was short and clear.

At the first workshop in School B with staff, the group suggested that no more than six staff should sit on the action group but that this may depend on school size and capacity. Staff thought that it was feasible to allow pupils to revise rules, as they already followed similar pupil-led principles in their school council and found that pupil-led rule revision increased the likelihood of pupils following rules. Staff reported that they also had an existing SEL curriculum and that an additional curriculum would not be feasible for primary schools. The assistant head confirmed that all staff had been trained in restorative approaches but that they would benefit from additional training to hold restorative conferences.

In the second workshop, staff agreed that online restorative-practice training was feasible as was all staff attending the two-hour introductory training. However, they thought that four or five staff attending the in-depth training was challenging. Staff suggested that that action-group members could communicate their involvement to the rest of the school using assemblies and badges or high-vis vests. They emphasised the importance of telling parents about the intervention goals and actions.

In the final workshop, staff agreed that the action group should include pupils from years three to six to gain wider perspectives but that younger pupils would not engage. They approved of the recruitment poster but suggested this be complemented by an assembly to introduce the programme to the school. The assistant head confirmed that pupils take minutes in their school council and suggested minutes could be used to share progress with other pupils. They also suggested assigning other roles to pupils to encourage participation. When handed copies of the draft manual, staff confirmed it was too complex for pupils to understand or engage with. However, they suggested providing a pupil-friendly version. Staff agreed that the proposed agendas in the manual were feasible. They thought that interim meetings without the facilitator would be beneficial, as would ‘catch-up’ calls between the facilitator and group chair, to plan meetings. Staff suggested that a senior-leadership-team member could chair the action group and lead the intervention but that the workload should be minimal and supported by another staff-member on the action group with behaviour-management experience. Staff thought pupils would understand the needs reports even if only presented as a table. They suggested that a pupil with good maths ability could guide their peers through the data.

***Online pupil workshop.*** Two workshops were held online with pupils from four primary schools on 25 ^th^ June and 8 ^th^ July 2024. Pupils from the fifth school attended separate workshops on the 1 ^st^ and 9 ^th^ July 2024. See Table 3 for pupil characteristics.

**Table 3.** Online workshop pupil demographics.

School	Year-group			Gender	
	*4*	*5*	*6*	*Girls*	*Boys*
1	0	2	0	1	1
2	0	2	0	1	1
3	0	0	3	2	1
4	2	0	0	1	2
5	0	0	2	1	1

During the first workshops, pupils discussed restorative practice. Pupils from all schools thought their school dealt with conflict in a restorative approach. There was widespread support for this approach. One pupil argued that punitive approaches cannot solve conflict and that, instead, pupils should be encouraged to consider how they might behave next time, taking into account each other’s perspectives. This emphasis on hearing both sides of the story was echoed by pupils from another school. They said that staff listening to only one pupil results in those pupils who were not listened to having negative feelings, and that punishment leaves pupils feeling frustrated, likely to harm again, or unable to concentrate in class. Pupils agreed that it was better to solve problems collaboratively. They felt that restorative practice might help perpetrators understand how their behaviour had impacted on another pupil, so they can change future behaviour. Several pupils also felt restorative practice would make dealing with bullying easier for teachers. However, one pupil criticised restorative practice, believing that not punishing a perpetrator was wrong and that pupils should be separated rather than brought together. Some pupils worried that restorative practice might spark further arguments. While some pupils were concerned perpetrators might lie or fake contrition, they thought this was less likely if perpetrators were encouraged to understand the harms they had caused.

In the second online workshops, pupils discussed action groups. Most supported these and would want to participate, with one pupil suggesting this might help pupils feel better about themselves. Some pupils worried that decision-making involves responsibility and the possibility of making mistakes. However, most saw this responsibility more positively. Some pupils emphasised the value of an opportunity to voice their opinion if something was unfair or needed to be changed. Several agreed that pupils would provide insights through their experiences that teachers might not know about. Pupils said they would feel comfortable working with pupils from other year-groups and with staff, providing there was a balance between staff and pupils. They supported group diversity and emphasised that everyone should have their say. One pupil thought that girls and boys might contribute different perspectives on bullying. It was agreed that groups could involve years four to six with uncertainty about the involvement of year three. Pupils supported the group communicating their decisions to the rest of the school in assemblies, to ensure others were informed and could provide feedback. This could also build pupils’ confidence and skills in public speaking. Some worried that members might be nervous presenting in assemblies, and presentations in classrooms were proposed as an alternative.

In the workshop, an edited version of the recruitment poster was discussed. Pupils approved the wording and agreed it would encourage children to apply, but suggested making it shorter and adding colour or photos to make it more appealing. Pupils suggested placing the poster in areas visible to most pupils such as noticeboards, and on the school website for their parents to see.

Pupils agreed that it was important to take minutes so that the group could review and reflect on what was said in each meeting. When shown an adapted version of the needs report, pupils understood and discussed the information contained. They agreed that the data provided would usefully inform decisions. Pupils suggested sharing the data in an assembly to raise awareness about the action group and encourage pupils to oppose bullying.

***In-person workshop with parents.*** This was held in-person on 24 ^th^ July 2024, with five parents/carers who had children across four schools. The group was diverse by ethnicity and included four females and one male. An additional participant was interviewed separately. Parents highlighted that what constituted ‘bullying’ could be nuanced with children finding it difficult to distinguish bullying from ‘banter’. They stressed that parents and children spoke about ‘bullying’ without a clear, common understanding of what it means, suggesting the importance of learning about this at school.

Parents discussed the possible benefits of restorative practice, including encouraging children to share reflections on their behaviour and listen to each other, to avoid misunderstanding. Parents felt it was important for children to appreciate each side of the story when in conflict, which they thought might encourage mutual understanding and respect. Parents thought restorative practice might help children feel valued by providing a sense of control when finding solutions. They suggested it might be used preventatively. Parents noted possible challenges in implementing restorative practice with pupils with special educational needs and disabilities, and with pupils too young to understand the impact of their actions on others. Parents suggested that children find it hard to articulate their feelings and would need help from teachers do so. One parent who was a teaching assistant at a local school said that restorative practice often falls to such staff, especially when conflict arises during playtime. She noted that teachers may lack the time or understanding to deal with bullying, and can consequently ignore conflict when it arises.

Appendix 6 summarises all suggestions and how these were addressed in refining the intervention.

### Online school survey

A total of 49 schools (1.3%) responded to the survey, the results of which are reported in Table 4. Of staff responding, 96% reported that restorative practice was feasible and acceptable in primary schools. Two responded that it was ‘maybe’ feasible and acceptable, and none thought it not feasible or acceptable. Only one school (2%) reported their school did not already use restorative practice, with 69% confirming they used it often and 29% occasionally. Of schools reporting already using restorative practice, 43.8% reported having a large number of staff trained to use this, 27.1% reported having a small number of staff trained to use it and 29.2% reported having no staff thus trained.

**Table 4.** Online survey of primary schools.

Item	Response options	n (%) * responses (N = 49) ^a^
Staff role	Headteacher/principal	10 (20.4)
	Member of senior leadership team (or equivalent)	26 (53.1)
	Lead for personal, social, health and economic education (or equivalent)	18 (36.7)
	Teacher	5 (10.2)
	Teaching assistant	1 (2)
	Administrator	0
Restorative practice is a method of addressing conflict or misbehaviour by bringing together those involved to repair the harms done and improve relationships. Do you think this is in principle a feasible and acceptable approach to use in primary schools?	Yes	47 (95.9)
	Maybe	2 (4.1)
	No	0
Does your school already use restorative practice?	Yes, often	34 (69.4)
	Yes, occasionally	14 (28.6)
	No	1 (2)
Are any of your staff trained to use restorative practice?	Yes, a large number	21 (42.9)
	Yes, a small number	13 (26.5)
	No	15 (30.6)
	Missing	41 (85.4)
Actions groups are a method of involving a few student representatives and teachers in reviewing information on student needs and planning how to address these. Do you think this is in principle a feasible and acceptable in primary schools?	Yes	7 (14.6)
	No	1
	Missing	26 (53.1)
Does your school already use action groups, or a similar approach?	Yes	23 (46.9)
	No	8 (30.8)
If already used, is this supported with any training or other support? (N = 26)	Yes, training	10 (38.5)
	Yes, other support	8 (30.8)
	No	46 (93.9)
Does your school deliver social and emotional skills lessons to students?	Yes	3 (6.1)
	No	43 (93.5)
If delivered, is this taught to students in the following years? (N = 46)	Y2	42 (91.3)
	Y3	43 (93.5)
	Y4	44 (95.7)
	Y5	44 (95.7)
	Y6	14 (30.4)
If delivered, around how many lessons are taught in any one year? (N = 46)	Between 1 and 5	32 (69.6)
	More than 5	2 (4.4)
If delivered, what is the average duration/length of each lesson? (N = 46)	Under 15 minutes	12 (26.1)
	15–29 minutes	20 (43.5)
	30–44 minutes	12 (26.1)
	45 minutes or over	23 (51.1)
If delivered, is this a named curriculum? (N = 46)	Yes	22 (48.9)
	No	1
	Missing	27 (55.1)
How much would your school be prepared to pay for an effective bullying prevention intervention at your school?	Nothing	22 (44.9)
	Up to £500	0
	More than £500 but less than £1000	0
	More than £1000	1
	Missing	19 (38.8)
Do you think it is feasible to ask primary school students to complete questionnaires themselves (about how they feel about school, their wellbeing, and experiences with bullying) if the questions use simple language and a researcher reads out questions and possible response options with the students?	Yes for Y1–Y6 students	10 (20.4)
	Yes for Y2–Y6 students	14 (28.6)
	Yes for Y3–Y6 students	4 (8.1)
	Yes for Y4–Y6 students	2 (4.1)
	No for all students	27 (55.1)
Would your school be interested in participating in a study of an intervention to prevent bullying if offered for free?	Yes	18 (36.7)
	Maybe	4 (8.2)
	No	

^a^ N = 49 unless otherwise stated in column “Item”.

^*^ All percentages calculated over non-missing values.

Regarding action groups, 41 (85%) thought it feasible and acceptable to involve pupils and teachers in groups which reviewed information on pupil needs, and planned how to address such needs. Seven schools (16%) did not think it was feasible. Of those who thought action groups were feasible, 25 schools responded that they already implemented something similar, with 16 schools responding that they did not. All seven schools who did not think action groups feasible reported not implementing them in their school. Of schools reporting using action groups or a similar approach, around one-third each reported receiving training, other support and no support.

Regarding the needs survey, a majority of schools thought it would be feasible to ask primary school pupils to respond to questions on their wellbeing and bullying experiences. Only two schools (4%) reported it would not be feasible to do this.

Almost all schools (96%) confirmed teaching an SEL curriculum to years two to six, with 70% reporting this included over five lessons per year.

Approximately half of schools reported being prepared to pay up to £500 for an effective bullying prevention intervention (45%) and just over half (55%) reported not being prepared to pay anything. No schools reported willingness to pay above £500.

### Adaptations

***Intervention theory of change and overall approach.*** No adaptations were made to the theory of change or overall approach based on our review of systematic reviews, PPIE and the online survey. The theory of change was informed by evidence from systematic reviews and the previous Learning Together trial as described above, and not challenged by the findings. In general, the review of systematic reviews, PPIE and online survey supported our existing plans.

***Social and emotional learning curriculum.*** We dropped the SEL curriculum from the overall intervention due to confirmation from both PPIE and our online survey that most primary schools already provide this and do not have capacity to introduce additional or alternative lessons. Our review of systematic reviews also found no evidence that SEL lessons are essential for intervention effectiveness.

***Restorative practice.*** Pupil and staff PPIE confirmed the relevance of delivering restorative practice in primary schools. We made very few changes since most comments aligned with our existing plans. Views from school staff, through both the workshops and the online survey, confirmed interest in incorporating restorative approaches in primary schools as well as the need for sufficient training and guidance to implement this correctly.

Following staff comments on primary school capacity, we decided to run the in-depth training on non-consecutive days to facilitate staff attendance and engagement, and requested that schools select between two and five staff. We were satisfied that this would allow for one-form schools to select fewer staff if needed, but would retain sufficient engagement and interaction during the training sessions and allow for bigger schools to select more staff.

***Needs survey and report.*** We retained the needs survey and needs reports. The needs report is a key component, enabling recentring school provision on pupil need and encouraging collaboration among pupils and staff, and was supported by most participants. Concerns about sharing information on free school meals and ethnicity were expressed by a minority of teachers. We decided to not present data by ethnic and free-school-meal sub-group for individual schools in the pilot trial, because of small and potentially identifiable numbers. However, we did present these sub-groups across all schools. Given pupils’ comments about girls and boys expressing different views, we retained stratification of needs by sex.

As most pupils struggled to understand the needs report without guidance, we edited the manual to suggest that action groups select one staff-member responsible for summarising key findings for pupils. The manual also now suggests encouraging pupils who easily understand the findings to explain the data to their peers. We decided to include pie charts in the reports, and the manual encourages the chair to re-present information which they find particularly pertinent for their school into visual format following comments suggesting incorporating visual representations of the data. Comments from pupils that they wished to see such data in order to inform decision-making confirmed our belief that it was important to provide a summary of pupils needs, albeit in a streamlined format. Given these adjustments, we felt satisfied that primary school aged children would be able to discuss and respond to data drawn from the needs reports.

Regarding the survey which informed these reports, we were unable to take on board all suggested changes due to the surveys drawing on standardised, validated questionnaire items. However, we produced a short guide for fieldworkers highlighting which words children were likely to find challenging. We also decided not to survey year-two pupils (aged 6–7), due to comments that they would struggle to complete the survey. This adaptation was greeted positively by staff when shared with them in subsequent workshops.

***Manual.*** The intervention manual was initially drafted informed by the previous Learning Together manual and was then informed by recommendations from the review of systematic reviews and PPIE. As a result of the review of systematic reviews, we added in the following to the manual as required elements of the work of school action groups: allocating specific and active roles to pupils on the action group during their first meeting (which also was recommended through PPIE); being clear how intervention components are synergistic; explaining how the intervention aim aligns with national policy requirements; requiring that action groups develop or refine school policies on bullying or discipline; and communicating the aims and contents of the intervention to parents via meetings or other communication methods deemed appropriate by the school (also recommended through PPIE). We also added the following as ideas for action groups to consider implementing: identifying hot-spots for bullying and increasing staff presence and visibility in these; developing or refining reward systems for good behaviour; and providing additional pupil activities at breaktimes.

Informed by the review of systematic reviews, we also included in the manual the need to ensure that restorative practice methods are tailored to school contexts informed by assessment of pupil needs and school characteristics. We decided to encourage schools to use the needs assessment reports to plan how they will implement restorative practice in their schools. We added in guidance that, where restorative practice works with children involved in bullying or other conflict, the classroom teachers of these pupils should be involved in the process. We decided not to add peer mediation as an intervention component because to do this well would require a quite different approach to the intervention involving the training of pupils in peer mediation. We were also cautious about the feasibility of peer mediation led by primary-school pupils.

In response to multiple comments from PPIE that pupils should be provided with an age-appropriate version of the manual, we edited and produced a simplified version, to be distributed to pupils in the action group. We felt that this would empower pupils to engage in the group. Pupils in PPIE expressed support for action groups to centre on empowering pupil voice in decision-making. This confirmed the need to ensure a good balance between pupils and teachers on the action groups, and facilitate discussion between these. Taking into account PPIE findings on primary school capacity, we decided to allow schools to select between two and six staff-members while encouraging them to select six where possible, and asked schools to select eight, rather than six, pupils. This was amended in the manual. We also edited the suggested age-range of pupils, and emphasised that, while schools must include years five to six, they could include younger pupils if they judged this appropriate.

The strong support for diversity within action groups confirmed the need to emphasise the importance of selecting pupils diverse in terms of age, gender, ethnicity, behaviour, and academic engagement and attainment. While this did not require adapting the manual as it was already included in our plans, we decided that it was particularly important to emphasise these requirements when communicating with schools.

Informed by pupil feedback, we edited the recruitment poster by simplifying the language and adding some suggested sentences to increase interest, such as “your opinion matters!” and “are you interested in bringing about change in your school?” However, we decided it was not appropriate to include colour images because schools may find it difficult to print in colour.

Although collaboration between pupils in different year groups and staff was overwhelmingly viewed positively, some pupils expressed concern that participating in the group would be intimidating. We therefore decided to add the discussion of ground rules (e.g., respecting others’ opinions within the group) to the first agenda in the manual, to ensure that pupils felt comfortable expressing themselves. We also highlighted in the pupil manual that pupils could decide on ways of communicating to their peers, for example by presenting to classrooms as well as holding assemblies. We adapted the suggestion that pupils be responsible for minutes taking to suggest they could do so alongside or supported by a staff-member.

Following staff feedback, we decided to encourage schools to hold interim meetings without the facilitator in addition to the obligatory termly meetings with the facilitator. As discussed with staff, we suggested that sessions with the facilitator could focus on deciding actions, while interim sessions could focus on following up on actions and monitoring progress. The group could then feedback to the facilitator at the next termly meeting. In response to staff comments that communication between the school and facilitator was vital for the groups to run well, we introduced conversations between schools and the facilitator before each termly meeting to help strengthen relationships and help schools plan for subsequent meetings. Finally, due to feedback concerning smaller staff capacities in primary schools compared to secondary schools, we decided that the school day-to-day lead and the action group chair could be the same person, and amended the manual accordingly.

## Discussion

### Summary of key findings

We adapted Learning Together for use in primary schools and elaborated our initial plans to generate an intervention supported by full materials, training and external facilitation. In line with this, we retained key components and did not modify our theory of change. The review of systematic reviews provided useful evidence which enabled us to identify and build on an evidence base for the intervention being adapted as recommended in the ADAPT guidance. ^29^

The PPIE and online survey enabled an exploration of the differences between secondary and primary schools as contexts for implementation, which was also recommended in this guidance. The online survey confirmed while many schools already implement restorative approaches, they lack adequate training to do so, and that an acceptable intervention must be free or low-cost. However, these findings may be unrepresentative of schools across England as the response rate to the online survey was low.

PPIE proved valuable in informing adaptation of Learning Together for primary schools in terms of ensuring its feasibility and acceptability. PPIE participants were well placed to comment on most aspects of our planned intervention. Teachers and senior leadership team members were well placed to comment on the likely feasibility of LTPS in their schools. Overall, pupils, staff and parents understood and were positive about the intervention and its application to primary schools. The PPIE allowed us to avoid potential unintended consequences of adaptation not taking adequate account of these differences, for example in terms of the very different size and capacity of primary schools, which meant that not so many staff can attend training or sit on action groups, and the very different abilities of primary-school-aged children, which affects how we drafted our needs reports, and how we recommended these be communicated to pupils. We engaged with these schools in a sustained and iterative way as recommended by the ADAPT guidance. We include in this paper an indication of how we meet the demands of the ADAPT reporting checklist for adaptation studies (Appendix 7). The final design of the intervention is presented in Appendix 8.

### Limitations

The review involved a rapid review of existing systematic reviews rather than undertaking a new systematic review of primary studies or a systematic review of reviews. It did not aim to synthesise the evidence in order to develop generalisable findings but instead aimed to extract useful insights from reviews in order to inform adaptation of Learning Together for primary schools. Many reviews were opaque in terms of reporting intervention details and many did not summarise the evidence particularly relating to the whole-school interventions delivered in primary schools to prevent bullying. We consulted the primary studies cited for more details where necessary to inform adaptations.

Our PPIE involved intensive and sustained engagement with the staff and pupils from two schools and was extremely useful in informing intervention adaptation. It was not research and did not aim to develop generalisable findings. Our online survey of primary schools had an extremely low response rate. We had reservations about including such a survey in our adaptation phase but included this element following a funder request informed by reviewer comments. These reservations were confirmed by the low response rate, suggesting such surveys are unlikely to be a useful component of future PPIE with schools. While the findings can in no way be considered representative of other primary schools in south-east England, they were nonetheless useful in confirming the findings from PPIE. For example, that a SEL curriculum was unlikely to be feasible or useful and that schools would value training in restorative practice.

### Implications for policy and research

It is possible to adapt an intervention originally used in secondary schools for delivery in primary schools drawing on evidence review, PPIE and surveys. PPIE is extremely useful in addressing questions of feasibility and acceptability.

Not all suggestions from the PPIE could or should be accepted and acted upon. It can be challenging to decide how to consolidate findings from PPIE with those from evidence review. Neither providers nor affected populations will necessarily have expertise in questions of intervention effectiveness so it is better to draw on research evidence to examine such questions. However, those with lived experience of providing or receiving interventions will have expertise in what factors need to be considered when adapting interventions for new settings and populations.

As with some previous analyses of public participation in health decision-making, we recommend that PPIE involves more open dialogues between researchers and PPIE stakeholders. Researchers and PPIE stakeholders should feel able to probe and challenge each other’s views, and examine what light evidence can shed on the questions being discussed. PPIE would also benefit from sustained engagement so that, where recommendations are not accepted, this can be reported back to participants and further discussed if necessary. Future work will include a feasibility study to optimise the intervention and assess whether progression to a full trial is justified.

#### Ethical statement

The research was conducted in accordance with the World Medical Association Declaration of Helsinki. Regulatory approval for the overall research project was given by the research ethics committees of London School of Hygiene and Tropical Medicine (reference 29958). Participants consented to the online survey research via an online form. The PPIE subcomponent of the overall study was not research, and therefore was not within the remit of the ethics committee. PPIE members were given verbal information about the workshops and freely agreed to participate. No PPIE sessions were recorded other than via written notes, which did not attribute any statements to individuals.

## Data availability

The consent materials for the online survey of primary school staff state that we will not share the data with anyone other than with coinvestigators at other institutions. We therefore do not have participants’ consent to upload the anonymised survey data on a public repository. The paper is also informed by patient and public engagement (PPIE). PPIE is not research and does not involve data. Any requests for access to research data should be addressed to the principal investigator, Professor Chris Bonell ( chris.bonell@lshtm.ac.uk). Access will be granted where the request is for a reasonable use of the data supported by an analytical protocol and ethics approval, and where the requested use does not contravene the conditions described above in relation to participant consent.

### Extended data

Open Science Framework: Public engagement, evidence review and survey to adapt a whole-school intervention to prevent bullying in English primary schools, 10.17605/OSF.IO/58KCU ^55^

This project contains the following underlying data:

• Appendix 1_Systematic reviews of which we were already aware and experts consulted.docx
• Appendix 2_Data extraction tools for review of systematic reviews.docx
• Appendix 3_Online survey.docx
• Appendix 4_Search results.docx
• Appendix 5_Information provided by included systematic reviews.docx
• Appendix 6_PPIE suggestions.docx
• Appendix 7_ADAPT reporting checklist.docx
• Appendix 8_LTPS design as piloted.docx
•
Figure 1_Logic model.jpg
• Supplementary Materials 1_consent form for online survey.docx

Data is available under CC0 1.0 Universal license.
