# Supplementary material for: Public engagement, evidence review and survey to adapt a whole-school intervention to prevent bullying in English primary schools
Source: NIHR Open Res. 2026 May 5;5:73. Originally published 2025 Sep 3. [Version 2] doi: 10.3310/nihropenres.13975.2 (PMC12743219; doi:10.3310/nihropenres.13975.2)
Supplement: Peer review history

# Reviewer report: Reviewer response for version 1

**DOI:** 10.3310/nihropenres.15191.r38451

**Published:** 2025-12-26

**Authors:** Caitlyn Donaldson

**Affiliations:** 1 Cardiff University, Cardiff, Wales, UK

Thank you for giving me the opportunity to review this paper. It is well written and an important contribution to school health intervention. The research questions are clearly defined and the methods clearly described.

 I have only a few suggested amendments.

**Introduction**

 In the first paragraph, can you add a reference to the Learning Together intervention and also a reference to whole school approaches.

**Methods**

 Line 5, you state that “UK” primary schools lack effective bullying prevention interventions based on PPIE conducted in England-only. Can you either provide evidence for other parts of the UK or change UK to English.

 Figure 1 – logic model. Can you provide a more detailed title for the figure – what is it a logic model of? The logic model is also repeated in the appendices which is unnecessary.

**Results**

 Throughout the systematic review results section, you use the descriptors “some” and “several”. Could you be more precise and include the numbers of papers/percentages – e.g. “5/21 (24%) papers…” to give a better indication of the extent of the findings.

 In the online school survey section and Table 4, the percentages/numbers are, in places, incorrect. E.g. “Around one-third confirmed their school already used restorative practice”– in the table, it is 69% that use it often/29% occasionally. The next sentence refers to 41% having a large number of staff trained, 16% small number of staff trained, 12% no staff trained, which doesn’t add up to 100% - in the table the percentages are 85%, 27% and 31%, which also doesn’t add up to 100%. For the action groups, 41/49 = 84%, not 85% etc. Please check all the percentages/numbers in the text and table are correct and reflect each other.  Also in table 4, some of the % are missing where the n value = 1.

Is the work clearly and accurately presented and does it cite the current literature?

Yes

If applicable, is the statistical analysis and its interpretation appropriate?

Partly

Are all the source data underlying the results available to ensure full reproducibility?

Yes

Is the study design appropriate and is the work technically sound?

Yes

Are the conclusions drawn adequately supported by the results?

Yes

Are sufficient details of methods and analysis provided to allow replication by others?

Yes

Reviewer Expertise:

School mental health, quantitative analysis

I confirm that I have read this submission and believe that I have an appropriate level of expertise to confirm that it is of an acceptable scientific standard.

## References

[1] : Public engagement, evidence review and survey to adapt a whole-school intervention to prevent bullying in English primary schools. *NIHR Open Research* .2025;5: 10.3310/nihropenres.13975.1 10.3310/nihropenres.13975.1 PMC1274321941458560

# Response

**Published:** 2026-04-13

**Authors:** Rose Eagle-Hull

Thank you for your feedback. We have made the below changes:

 - Added a reference to the Learning Together intervention and whole school approahces

 - Edited line 5 to refer only to English primary schools

 - Provided a more detailed title for the figure ( **Figure 1. Logic model of LTPS theory of change**). NIHR required us to upload this image separately in the same online database as the appendices, however I appreciate that this is unnecessary duplication and will request for it to be removed from the online database if allowed

 - Added detail to the systematic review results 

 - Clarified denominators and missing numbers in the online school survey section to correct errors 

 Many thanks again for your comments.

# Reviewer report: Reviewer response for version 1

**DOI:** 10.3310/nihropenres.15191.r37490

**Published:** 2025-11-03

**Authors:** Emma Soneson

**Affiliations:** 1 University of Oxford, Oxford, England, UK

Many thanks to the NIHR for the invitation to review this paper by Eagle-Hull et al., which describes the adaptation of the Learning Together whole-school anti-bullying intervention for use in primary schools (Learning Together Primary Schools, or LTPS). The authors describe an adaptation process including a rapid review of published evidence; an extensive programme of patient and public involvement and engagement (PPIE) with pupils, school staff, and parents; and an online survey of primary school staff.

 My first impression in reading this manuscript was that I wished every intervention adaptation was completed and documented as clearly and carefully as LTPS is in this paper. The authors have taken a robust approach to adapting this intervention for a younger population, and I was very impressed by the thoroughness of their process and comprehensiveness of their reporting. A few things I particularly appreciated were:

- The careful planning that will have gone into this process. The research team’s expertise in school interventions is readily apparent from this manuscript, and I was convinced that they had thoroughly considered differences between primary and secondary school settings in adapting Learning Together.
- The robustness of the PPIE involved. I was particularly encouraged to see that schools were ‘asked to recruit relatively disengaged pupils’ (particularly as schools themselves needed ‘Good’ or ‘Outstanding’ OFSTED ratings). It’s so much easier to recruit pupils with high levels of engagement, but having this diversity will undoubtedly provide insights that might not otherwise have been apparent. If available, it would also be helpful to know whether any of these pupils had experience of bullying/victimisation.
- The clear description of how findings from the three strands (review/PPI/survey) informed (or not) adaptations. I particularly appreciated Appendix 6, which detailed findings, which strand(s) they came from, whether they were actioned, and how the intervention was changed on account of them.
- Inclusion of the logic model (Figure 1) and LTPS design overview (Appendix 8).
- Transparent reporting, informed by the ADAPT guidelines, with clear descriptions of elements such as the rapid review (e.g. that it was *not *intended to be a systematic review), the composition of the PPIE groups, the content of the PPIE meetings/workshops; and the limitations of the online survey.

On the whole, I am convinced of the rigour with which this adaptation process occurred and look forward to seeing results from the pilot trial.

 By way of suggestions for improvement, I have relatively minor comments:

1. **Online survey analysis: **Some of the figures in the *Online school survey *section seem to be off. For example, it says ‘Around one-third confirmed their school already used restorative practice’, whereas Table 4 suggests this figure is 98.0% (48/49). In the next sentence, it says ‘Of schools reporting using restorative practice often, 41% reported having a large number of staff trained to use this, 16% reported having a small number of staff trained to use it and 12% reported having no staff thus trained’, which makes me wonder what the other 31% have responded. In other places, the percentages seem ever so slightly off – reporting numerators and denominators here might be helpful for clarity (including around whether schools with missing data were included in the denominators). Slightly more detail in the *Online school survey *section of the Methods regarding analyses would also be helpful (right now there is no detail; it just says ‘Data were analysed using Stata’).
2. **PPIE findings: **Whilst I do appreciate the careful reporting around the PPIE, this section alone is nearly 3,000 words and became quite repetitive, especially where there were multiple groups of pupils and school staff. It would be helpful to consider whether there might be ways to reduce repetition, for example, through presenting all pupil findings together and all staff findings together, noting areas of agreement and disagreement as relevant. A table (in the main text) may also help to summarise learnings and identify where there was intra- and inter-group (dis)agreement (e.g. in whether to provide pupils with the manual, whether pupils could understand data in a needs report, whether it was appropriate to report aggregated data by protected/sensitive characteristics).

I also had a few musings that may or may not be of interest to the authors about the intervention itself and how it will be brought forward. First, the Discussion mentions that an acceptable intervention ‘must be cost-effective’. I didn’t read the survey as such – I read it as the intervention ‘must be free or low-cost’. Reading this, I wondered what implications this might have for future (cost-)effectiveness trials and wider implementation. Second, I would be interested to know whether the manual will have any guidance on how action groups should be selected (beyond ensuring diversity) – would pupils apply for these roles? Would schools choose? What happens in the case of oversubscription? Third, given wider evidence around statistical literacy (even in adults!), I remain unconvinced that primary school pupils will be able to meaningfully grasp percentages, even in pie chart format. I’d be really interested to see how that part of the work develops as the authors move forward, as the needs report seems so essential to the intervention. Anyways… just thoughts, and no direct action is needed with regards to this specific publication.

 Finally, I noted with interest that the survey was a requirement of the funder. With utmost respect for the NIHR, I think that the survey, particularly in the context of a 1.3% response rate, provided some of the *least *helpful information about potential adaptations and likely took quite a lot of effort. It might be useful in the future to consider whether these kinds of requests are merited.

 Thank you again for the opportunity to read this – I wish the authors all the best with their next steps!

Is the work clearly and accurately presented and does it cite the current literature?

Yes

If applicable, is the statistical analysis and its interpretation appropriate?

Partly

Are all the source data underlying the results available to ensure full reproducibility?

Yes

Is the study design appropriate and is the work technically sound?

Yes

Are the conclusions drawn adequately supported by the results?

Yes

Are sufficient details of methods and analysis provided to allow replication by others?

Yes

Reviewer Expertise:

Child and adolescent mental health; public health; school-based interventions

I confirm that I have read this submission and believe that I have an appropriate level of expertise to confirm that it is of an acceptable scientific standard.

# Response

**Published:** 2026-04-13

**Authors:** Rose Eagle-Hull

Thank you for your feedback. Please see below our response in order of your comments:

 We don’t know for sure whether the pupils involved in PPIE had experiences of bullying or victimisation. We didn’t think it would have been appropriate to query this in a group setting during PPIE as it could have triggered sensitive conversations and safeguarding issues. However the teachers recruiting pupils for PPIE did emphasise that they included ones who struggled with their behaviour and generally primary school teachers do have a fairly good idea of pupils who have challenges with bullying. Though we don’t know for sure exactly how many had challenges with bullying, we are reasonably confident of having captured these views.

 We have edited the table and text relating to the online survey analysis, and have included a note to explain different denominators and any missing values. We have edited the text in the methods to “. Descriptive analysis using Stata (StataCorp LLC, Version 15) was used to calculate frequencies.”

 Regarding the PPIE findings, thank you – we appreciate that this section was quite repetitive. We have decided to retain the overall structure in that the results are structured according to data source to avoid conflating findings from different groups. I have however gone through this section to reduce the word count.

 We agree that you make a good point regarding acceptable interventions should be low-cost rather than merely cost-effective and have edited this accordingly. 

 Regarding guidance on action group recruitment, schools were asked to select pupils to ensure diversity, whether by selecting pupils themselves or selecting from pupils who had applied. The manual included an example poster for schools to use to advertise the action groups, explaining that pupils could contact their teacher to apply to be involved. In reality most schools selected pupils themselves rather than advertising for pupils to apply. Roles are decided during the first Action group meeting, led by the chair. This is explained in the manual. I have edited the manuscript to make this clearer.

 Regarding the needs assessment report (NAR) and statistical literacy, in reality the pupils didn’t look at the NARs in the same table format that they are presented to schools. The AG chair went over the NAR with Place2be facilitator in advance and then showed a summary of the info to the students. This was usually presented with simplified tables/charts/diagrams or verbally, highlighting key findings. Interested pupils were given the option of looking at the pie charts. We have since included clearer guidance for teachers to select and present the information in the manual for a future phase III trial (reminding them that they only need communicate 1-4 important findings for the discussion to be meaningful, and guidance for translating percentages into simple terms).

 We agree with respect to your comments that the online survey was not very helpful and took a lot of effort. We had reservations about the survey when it was requested and these were confirmed. We have edited the discussion slightly to reflect this.

 Thank you very much for your feedback.
